# Safety surveillance for PrEP in pregnant and breastfeeding women

**DOI:** 10.3389/frph.2023.1221101

**Published:** 2023-09-29

**Authors:** Lee Fairlie, Diane Lavies, Emma Kalk, Otty Mhlongo, Faeezah Patel, Karl-Günter Technau, Sana Mahtab, Dhayendre Moodley, Hasina Subedar, Saiqa Mullick, Shobna Sawry, Ushma Mehta

**Affiliations:** ^1^ Wits RHI, Faculty of Health Sciences, University of the Witwatersrand, Johannesburg, South Africa; ^2^ Centre for Infectious Disease Epidemiology and Research, Faculty of Health Sciences, University of Cape Town, Cape Town, South Africa; ^3^ KZN Department of Health, Durban, South Africa; ^4^ Empilweni Services and Research Unit, Rahima Moosa Mother and Child Hospital, University of the Witwatersrand, Johannesburg, South Africa; ^5^ Wits Vaccines & Infectious Diseases Analytics (VIDA) Research Unit, University of the Witwatersrand, Johannesburg, South Africa; ^6^ Department of Obstetrics and Gynaecology, School of Clinical Medicine, College of Health Sciences, University of KwaZulu-Natal, Durban, South Africa; ^7^ Department of Health, Pretoria, South Africa

**Keywords:** pregnancy, breastfeeding, post-marketing surveillance, pre-exposure prophylaxis (PrEP), pharmacovigilance, teratovigilance

## Abstract

The risk of HIV acquisition is higher during pregnancy and postpartum than other times. Newly acquired maternal HIV infection associated with high primary viraemia, substantially increases the risk of vertical HIV transmission. Pre-exposure prophylaxis (PrEP) reduces the risk of HIV acquisition. Currently available products include oral tenofovir/emtricitabine (TDF/FTC) and tenofovir alafenamide (TAF)/FTC), long-acting cabotegravir (CAB-LA) and the dapivirine ring (DVR). All except oral TDF/FTC have limited safety data available for use in pregnant and breastfeeding women. The safety of new PrEP agents for pregnant women and the fetus, infant and child, either exposed *in utero* or during breastfeeding is an ongoing concern for health care workers and pregnant and breastfeeding women, particularly as the safety risk appetite for antiretroviral (ARV) agents used as PrEP is lower in pregnant and breastfeeding women who are HIV-uninfected, compared to women living with HIV taking ARVs as treatment. With the widespread rollout of TDF/FTC among pregnant women in South Africa and other low-middle income countries (LMIC) and the potential introduction of new PrEP agents for pregnant women, there is a need for safety surveillance systems to identify potential signals of risk to either the mother or fetus, measure the burden of such a risk, and where appropriate, provide specific reassurance to PrEP users. Safety data needs to be collected across the continuum of the product life cycle from pre-licensure into the post-marketing period, building a safety profile through both passive and active surveillance systems, recognising the strengths and limitations of each, and the potential for bias and confounding. Pharmacovigilance systems that aim to assess the risk of adverse birth outcomes in pregnant women exposed to PrEP and other agents need to consider the special requirements of pregnancy epidemiology to ensure that the data derived from surveillance are sufficiently robust to inform treatment policies. Here we review the known safety profiles of currently available PrEP candidates in women of child-bearing potential, pregnancy and breastfeeding and discuss pragmatic approaches for such surveillance in HIV-endemic LMICs.

## 1. Background

Young cisgender women in high HIV-burden countries remain at substantial risk for HIV acquisition: Birdthistle et al., estimated a pooled incidence of 5% in 20–24 year olds in a systematic review and meta-analysis that included data from 10 high prevalence African countries (1). Another study estimated a 10% incidence of HIV infection in 15–24 year old women in 15 high prevalence sub-Saharan African (SSA) countries between 2015 and 2019 (2). Risks of HIV acquisition during pregnancy and breastfeeding are also extremely high. In cisgender women in sero-different relationships in 7 countries in Southern and Eastern Africa, HIV incidence per hundred person years was 1.25 (CI 95% 0.95–1.62) in non-pregnant women, 3.75 (CI 95% 1.22–8.75) in early pregnancy, 7.02 (CI 95% 3.74–12.01) in late pregnancy and 4.68 (CI 95% 1.72–10.18) postpartum (3). Similarly, a recent systematic review and meta-analysis conducted in SSA, estimated HIV incidence at 3.6 per 100 person years (95% PI: 1.2–11.1) during pregnancy and breastfeeding combined (4). These high rates of maternal HIV infection increases the risk of HIV transmission to the fetus or baby during pregnancy, delivery, or breastfeeding. The transmission risk is especially high when the woman is unaware of her HIV status, is not yet receiving antiretroviral therapy (ART) as treatment, and has a high HIV viral load (5). High rates of HIV acquisition among young women of child-bearing potential (WOCP) and among women who are pregnant or breastfeeding, highlight the importance of including these groups in HIV prevention programmes.

Significant progress has been made in the development of biomedical products for prevention of HIV infection. The incidence of new HIV infections in pregnant and breastfeeding women, as well as adolescent girls and young women (15–24 years), has declined (although at a slower rate compared to adolescent male counterparts) with Graybill et al., reporting HIV incidence of 4.1 per 100 person years (95% PI: 1.1, 12.2) pre-2010, compared to 2.1/100 person years (95% PI: 0.7, 6.5) post-2014 (4, 6, 7). Several studies have clearly demonstrated the efficacy and acceptability of pre-exposure prophylaxis for HIV (PrEP) in pregnant as well as non-pregnant and breastfeeding women (8–15). National HIV programmes globally are implementing policies that include the rollout of products for the prevention of HIV in all populations considered to be at risk, including WOCP, pregnant and breastfeeding women (16–18). Currently the World Health Organization (WHO) recommends three products for PrEP – (i) oral tenofovir (TDF)/emtricitabine (FTC) PrEP available in fixed dose combination (FDC), (ii) the dapivirine vaginal ring (DVR) and iii) intramuscular long-acting cabotegravir (CAB-LA) (16–18). There is a pipeline of new agents that could be used for HIV prevention in pregnant women including oral/subcutaneous lenacapavir, antiretroviral-containing vaginal films and subcutaneous patches, and new oral antiretrovirals such as TAF/FTC. A well-established surveillance system would support the safe introduction of these products in pregnant women and WOCP as they become available. Specific safety indicators that clinical trials may not collect in a large enough sample, but surveillance systems would be able to collect include congenital anomalies, pregnancy and birth outcomes (stillbirth, prematurity, birth weight, neonatal mortality), exacerbation of pregnancy-related conditions such as gestational hypertension, gestational diabetes mellitus and child health outcomes including growth, neurodevelopment and malignancies (19). We summarise available safety data in PrEP agents in WOCP, pregnant and breastfeeding women, as well as discuss in detail approaches to surveillance in this population, specifically related to PrEP rollout.

### 1.1. Data on PrEP in pregnant and breastfeeding women

Oral TDF/FTC, which has an estimated efficacy of 97% in cisgender women when taken as prescribed, has been recommended by WHO since 2017 in pregnancy and breastfeeding, to complement other HIV prevention mechanisms in women at “substantial risk” of HIV infection (16, 17). Initial safety data were based on the use of TDF/FTC in combination with other ARVs in women living with HIV (WLHIV), requiring it for treatment, and later, in women without HIV receiving TDF/FTC as preventative therapy during pregnancy and breastfeeding (20–24). Published clinical trials and systematic reviews report reassuring safety data with minimal concerns regarding the use of TDF/FTC in pregnancy/breastfeeding for women or/and their infants (8, 20, 25–27). However, common side effects include gastrointestinal symptoms (nausea, vomiting, loss of appetite), headache and rashes, all of which may compound common pregnancy-related symptoms. In addition, elevated creatinine and subsequent renal damage may occur rarely, as well as reduction in bone mineral density (28). The PRIMA study in Kenya found that oral TDF/FTC uptake was higher in pregnant women at higher risk of HIV acquisition. Adherence was higher in pregnancy compared to postpartum and having a partner with a known HIV infection was the most significant predictor of initiation, adherence, and continued use. Interestingly tolerance to ARVs side effects was not an important predictor of adherence (9). The PrIYA program in Kenya evaluated pregnancy outcomes in 1530 mother-child pairs in Kenya, including 206 women who initiated TDF/FTC pre-conception compared to 1,324 with no TDF/FTC exposure (29). No increased rates of prematurity or low birth weight were seen in the TDF/FTC-exposed group, there were no congenital anomalies reported in the TDF/FTC-exposed group (5 in the PrEP-unexposed group) and at six weeks of age growth was similar in both groups (29). A study from Durban, South Africa, compared immediate initiation of TDF/FTC in pregnant young women, to deferred initiation post breastfeeding cessation, and found no increase in prematurity or low birth weight infants in those women receiving TDF/FTC during pregnancy (14). In addition, very low TDF/FTC concentrations are secreted in breast milk (30). Data from infants exposed to maternal TDF/FTC compared to those unexposed, from Maternal-Child and Vertical transmission programmes in Kenya, showed no differences in infant anthropometry at birth, 6- or 9-months (10).

Increasingly, country-based National Programmes are offering TDF/FTC to WOCP, pregnant and breastfeeding women, although roll-out progress remains slow, particularly in some high HIV-burden countries (12, 31).

The Dapivirine Vaginal Ring (DVR) which contains 25 mg of dapivirine, a non-nucleoside reverse transcriptase inhibitor, has been studied in cisgender women in SSA, and was shown to reduce risk of HIV infection by 27%–35% in clinical trials and by over 50% when adherence to the product is optimal (32, 33). Side effects may include cervical inflammation, reddening or swelling; urinary tract infections as well as bladder control problems; headache, pelvic pain and pain during sex, although all were rare and of mild severity in clinical trials (26). Data in pregnancy and breastfeeding are limited, but the Microbicide Trial Network (MTN) is conducting studies specifically focussed on these populations. The MTN 016 study evaluated pregnancy outcomes in 169 women who conceived while using the DVR on study (179 incident pregnancies) and discontinued the product when their pregnancy became known, at median gestational age of 5.4 weeks. In this study there were 105 (58%) full-term live births, nine (5%) preterm births, 39 (22%) spontaneous abortions, 22 (12%) elective abortions, four (2%) stillbirths and eight (7%) congenital anomalies (all minor). There was no statistical difference in pregnancy outcomes between the DVR and placebo arms (34). The ongoing MTN 042/DELIVER (NCT03965923) study is evaluating safety and acceptability of the DVR in pregnant women, beginning with the enrolment of women with more advanced gestational age: 3rd trimester in cohorts 1 (> 36 weeks, *n* = 148) and 2 (30–35 weeks, *n* = 154) respectively, and 2nd trimester in cohort 3 (12–29 weeks). Data from cohorts 1 and 2 have been published and showed low rates of pregnancy complications. Hypertension in pregnancy was the most common adverse outcome and there was one stillbirth and one neonatal death in each cohort, balanced across arms (35). Premature delivery occurred in 2% of cohort 1, and 6% of cohort 2 (35). To provide background pregnancy outcome rates as a comparison, MTN 042B, which was a cross sectional systematic chart review, was conducted in the same sites as MTN 042/DELIVER. Adverse outcomes in MTN 042/DELIVER were similar to background rates in MTN 042B for cohorts 1 and 2 (36, 37). However, in MTN 042/DELIVER, participants were enrolled late in pregnancy and carefully screened to exclude those with increased risk of prematurity and other potential complications. The study is currently fully enrolled, and all delivery outcomes completed by mid-2023. The MTN-043 B-PROTECTED (NCT04140266) study that enrolled postpartum women exclusively breastfeeding, also reported no safety concerns and minimal maternal systemic detection of dapivirine on pharmacokinetic measurement. There was negligible secretion of dapivirine in breastmilk and in infant pharmacokinetic sampling (38). Although efficacy of DVR is lower than TDF/FTC and CAB-LA, the advantage of providing choice of product to pregnant/breastfeeding women, particularly since extremely low exposure to product occurs for the fetus, is potentially compelling. DVR is registered in several high HIV-burden countries in SSA, including South Africa, Zimbabwe and Zambia (39). Due to the high cost, rollout in routine public health settings beyond demonstration projects (implementation science projects where products not yet readily available in public facilities are made available to enrolled participants) is not yet under consideration. Demonstration projects in SSA countries are planning to deliver DVR to WOCP in 2023, some of whom may become pregnant. Although the product is not registered for pregnant/breastfeeding women yet, and pregnant women will not be enrolled currently, women participating in the demonstration projects, who become pregnant while using DVR may have the option to continue using the product should they wish to, although this is project-dependant.

Long-acting cabotegravir (CAB-LA) is an integrase inhibitor, administered intramuscularly 2-monthly, which showed an 88% lower HIV acquisition risk in young cisgender women compared to TDF/FTC in the HVTN 084 study (40). Side effects may commonly include localised reactions at the injection site, as well as gastrointestinal side effects, sleep disturbances including abnormal dreams, anxiety and tiredness (26). The HVTN 084 study did not specifically enrol pregnant women, and CAB-LA was withheld if participants tested positive for pregnancy. Participants who became pregnant while on the study were offered the option of switching to TDF/FTC for the duration of the pregnancy. In 27 women who became pregnant while receiving CAB-LA, compared to 18 women who conceived on TDF/FTC, there were no significant differences in pregnancy or infant outcomes between the two groups (41). Pharmacokinetic (PK) drug levels measured by apparent terminal phase half-life were similar in pregnant and non-pregnant women. Because CAB-LA was stopped once pregnancy was diagnosed, PK levels from the 2nd or 3rd trimester were not evaluated (41). Given the long half-life of CAB-LA, even in women who have stopped treatment pre-pregnancy, the product may still be classified as active treatment and exposure during pregnancy, which has consequences for surveillance (42). Open-label extension studies, due to start in 2023, as well as upcoming demonstration projects, will allow continuation of CAB-LA during pregnancy, if desired by the participant, providing an opportunity to collect much-needed safety data on CAB-LA's safety in pregnancy CAB-LA is currently registered in a number of countries including South Africa, Botswana, Zambia and Zimbabwe but, due to the high cost, rollout in routine public health settings beyond demonstration projects is not yet under consideration (39).

## 2. The importance of sufficient robust PrEP safety data in pregnancy and breastfeeding

Whilst the increasing availability of PrEP products provides hope for prevention of HIV infection in WOCP, pregnant and breastfeeding women, safety data in pregnancy and breastfeeding remain limited and inadequate. Arguably, the greatest concern with respect to both fetal and maternal risk is during pregnancy, since breastfeeding women return to pre-pregnancy metabolism within about 6 weeks post-delivery, and as long as limited product is secreted in the breastmilk, which is largely the case with the products described above, there is less concern regarding safety during lactation (29). Clinical trials are essential to provide data regarding product dosage, pharmacokinetics, efficacy, safety, and acceptability. Pregnant women are generally excluded from these early phases of drug development and data, particularly regarding efficacy can be extrapolated from adult trials in non-pregnant individuals. In terms of safety, clinical trials are usually too small and too short to provide adequate data to understand the real risk-benefit profile of a product. In clinical trials involving pregnant women only reasonably healthy participants with no or well-controlled co-morbidities, and no preceding pregnancy complications, or risks for complications such as multiple gestation are included. Additionally, adolescents are frequently excluded from clinical trials and may be at higher risk of adverse complications, as well as more likely to access PrEP, therefore the absence of data is problematic (44). Moreover, in pregnant populations, different pharmacokinetics and possibly pharmacodynamics, and the presence of a developing fetus expands the scope of safety assessment of products such as PrEP. Populations in which PrEP will be used are far more diverse than clinical trial participants, use the product for longer periods than the duration of clinical trials, and use products in combination with other comorbidities and medicines which are often excluded in the clinical trial population (19).

**Figure 1:**
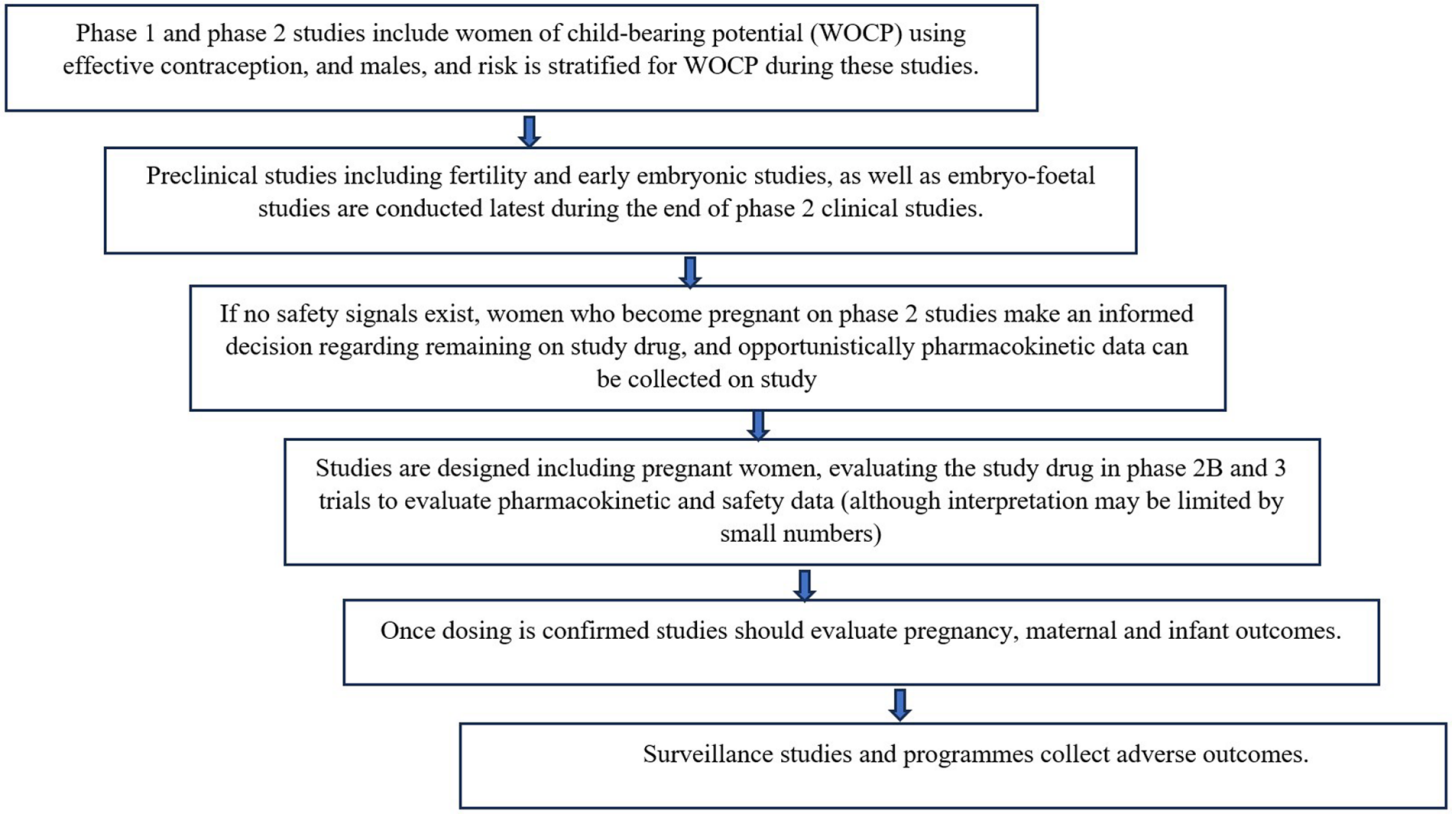
Framework for the safe inclusion of pregnant women in clinical trials.

Frameworks for safe approaches to inclusion of pregnant women as early as possible in clinical research have been developed (19, 43). Proposed steps include the following:

Figure 1 describes the framework that could be used for evaluation of new drugs, including PrEP, in pregnant women (34, 35).

Common pregnancy and birth adverse outcomes such as prematurity, small for gestational age, hypertension etc. may occur with high enough frequency to require a smaller sample sizes to detect a significant difference in risk of these outcomes. However, outcomes such as specific birth defects, which occur far less frequently, require much larger sample sizes to detect a significant difference in risk of these outcomes (45). For example, if overall congenital disorders occur at around 3%, at least 200 exposures are required to exclude a 2-fold increase in risk, whereas specific rare anomalies such as neural tube defects (0.1%) require at least 2000 exposures during the gestational period of risk to exclude a 3-fold increase in risk (45). Clearly, clinical trials are unlikely to enrol such numbers, nor should they, as requiring such large studies would further delay registration and availability of necessary products for pregnant women. Therefore, all steps in the framework, culminating in surveillance, are important.

**Figure 2:**
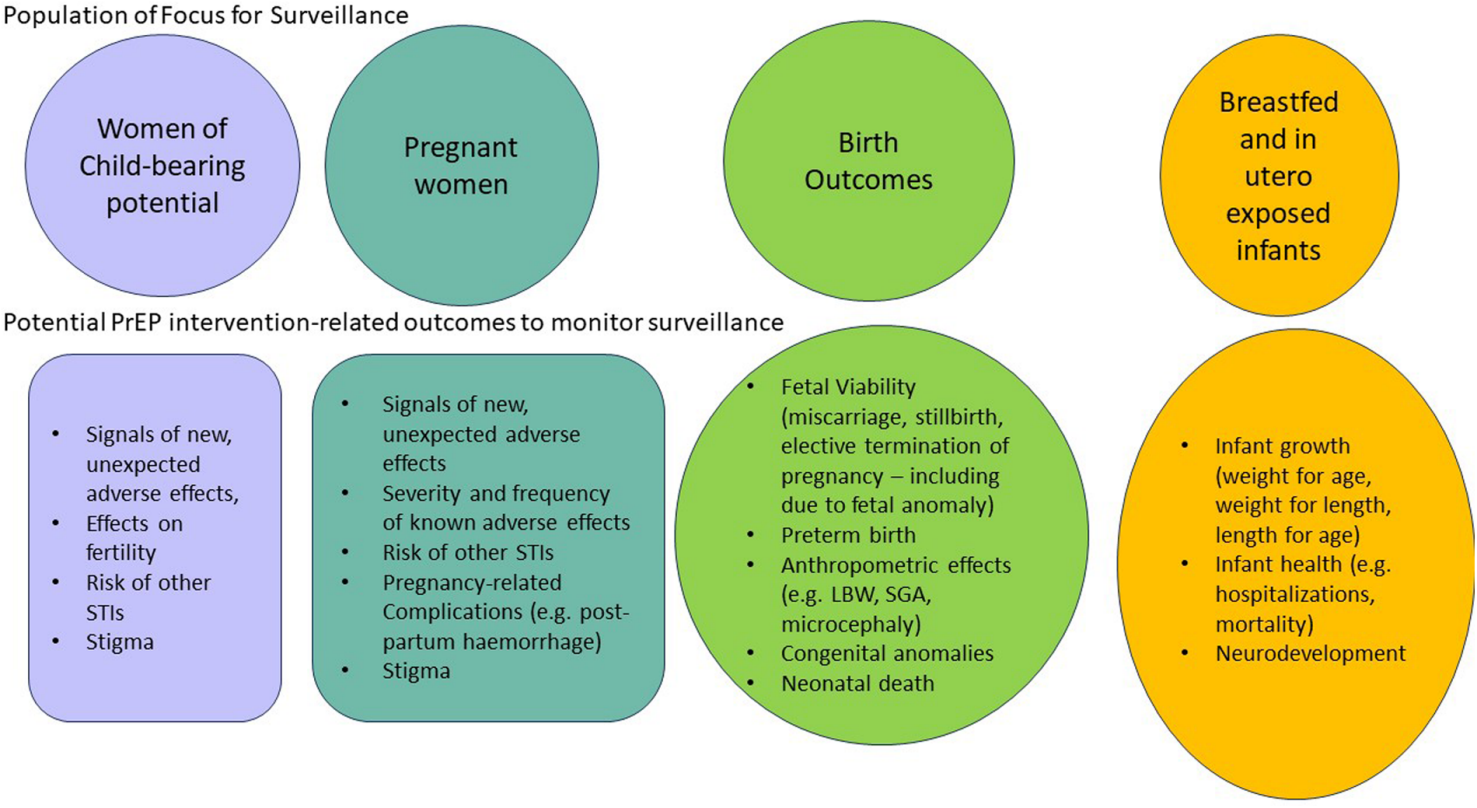
Populations and related key safety-related outcomes to include in surveillance of Pre-exposure prophylaxis (PrEP) products.

Surveillance systems need to be able to identify signals of adverse events not detected in clinical trials that are associated with the use of PrEP products. WOCP, pregnant women and their products of conception and children exposed *in utero* or during breastfeeding are the key populations to include in any PrEP safety surveillance plan. Each of these exposed populations have specific outcomes that should be monitored for in terms of the risk of PrEP exposure. Figure 2 provides an overview of key safety-related outcomes that need to be assessed as part of any PrEP rollout plan. Ensuring that key safety outcomes are adequately and systematically assessed is particularly pertinent for HIV*-prevention* products, where the threshold of acceptable risk may be lower than with HIV*-treatments* in which benefits are proven. In contrast, in situations where pregnant women require specific ARV regimens to treat a resistant HIV virus, the risk/benefit balance may sway towards treating HIV in order to achieve virological control, improve maternal health and prevent HIV infection through vertical or horizontal transmission, even if the required therapy has minimal safety information in pregnancy. Similarly, if a more favourable HIV treatment drug becomes available and is rolled out to the general population, the risk/benefit balance may shift to encourage use in pregnant women, living with HIV even with limited safety information about the drug, as it would be potentially inequitable to exclude pregnant women. For example, in the Tsepamo study in 2018, a signal was detected for the increased risk of neural tube defects in babies born to mothers who conceived on dolutegravir, compared to WLHIV receiving other ART regimens and to women without HIV (46). An interim analysis initially reported an increased risk of 0.94% (compared to around 0.1% in WLHIV on other ART and HIV-negative women). However, the risk decreased to 0.11% in 2022 after repeat analyses including a larger number of women (47). Importantly, despite the initial, apparent increased risk, and the resultant recommendation to exercise caution with the use of DTG periconception, the benefits of DTG including a more favourable side effect profile, faster viral suppression and reduced risk of developing HIV resistance resulted in ongoing use and advocacy supporting its use even in pregnant women (49). This study highlighted the need for bridging studies when products are prescribed for pregnant and breastfeeding populations, as well as the need for epidemiologically robust surveillance of HIV treatment and prevention products in the post-marketing period.

## 3. Safety surveillance systems

Given that many PrEP products are new, and considering the approaches to safety surveillance in WOCP, pregnant and breastfeeding women described previously, surveillance systems are required to enhance and confirm available data from clinical trials, implementation trials and demonstration projects. These systems form the final tier in evaluating PrEP safety and there are numerous types of surveillance that can be implemented. Safety surveillance systems for PrEP need to incorporate both passive and active surveillance systems. Active surveillance systems must have the capacity to measure risk by reliably comparing collected rates of adverse outcomes of interest between exposed and unexposed populations whether these unexposed groups are contemporary or historic controls.

Passive surveillance systems are usually implemented nationally or across many countries and therefore provide an inexpensive option for the detection of signals of previously unknown or poorly understood adverse events. Active safety surveillance systems are best placed within environments where a high number of exposures are expected in WOCP or pregnancy, and specifically where the disease is common, or medication required for treatment or prevention is commonly used. For example, in South Africa where around 7.5 million people are living with HIV (50), active safety surveillance systems for HIV treatment and prevention are much needed. Unintended pregnancies are common, 33.9% overall and 55.9% in WLHIV in two pooled analyses from SSA, and as high as 71% in a recent study from Cape Town, South Africa in WLHIV (51–53). This increases the chance that conception on treatment may occur, even if a drug is not licenced in pregnancy. Although the high rate of unintended pregnancy needs to be addressed urgently, this provides an opportunity for surveillance and to ensure that signals for adverse outcomes are reported as early as possible and closely monitored.

Standardisation of key maternal, pregnancy, birth and infant outcomes to be measured in surveillance programs allows for data pooling with other national and international programs, where increased numbers of exposures across differing geographical locations and populations adds strength to ascertainment of exposure-associated risk/s provided that the methods for data collection are comparable. A component of antenatal care which frequently presents challenges, is accurate gestational dating. Calculated using early ultrasound, reliable last menstrual period, gestational age assessment at birth or fundal height, gestational dating is critical to ascertain the timing of exposure of interest as well as outcomes such as prematurity, distinguishing stillbirth from miscarriage based on gestational age, and estimating low-for-gestational-age birth weight (54).

Teratovigilance studies the exposure of the fetus to external factors (drugs, substances, environmental factors, etc) and any resultant foetal developmental abnormalities and their impact on public health using epidemiological approaches (55). Teratovigilance is not confined to structural malformation but includes effects such as fetal loss, preterm delivery, impaired fetal and infant growth and development (55). Often such studies are designed to assess the risk of specific teratogenic effects that may have a biological plausibility or hypothesis based on animal and human studies with the specific drug in question or from drugs in the same class. For instance, long term follow-up studies aimed at assessing the safety of integrase inhibitors may form a critical component of the rollout plan for CAB-LA given early signals of neurodevelopmental and neurological effects associated with integrase inhibitor use (56–58). In establishing teratovigilance systems, a number of factors must be considered. Although pre-clinical data may be reassuring, animal models do not reliably predict congenital anomalies in humans. Therefore, unexpected findings may arise and may require verification in other settings or populations. Teratovigilance systems need to be designed based on the key objectives of the system, the health-seeking behaviour of pregnant women in the communities where medicines of interest are commonly prescribed, the key risk drivers of the medicine/s being investigated and the threshold of acceptable risk for these medicines in pregnant women, considering their benefit profile. Classification systems such as EUROCAT and WHO have different categories for minor and major anomalies, resulting in a lack of uniformity across surveillance systems when reporting rates of congenital anomalies (59, 60). Usually, surveillance systems are only able to capture surface examination findings of congenital anomalies at the time of delivery. Internal congenital disorders such as cardiac, renal or other anomalies may only be detected much later on as well as longer term effects on growth and neurodevelopment. This is expected and needs to be noted as a potential limitation of such systems. Stillborn surface examination is difficult, depending on the state of the fetus, and may be inaccurate in macerated stillbirths. Health care workers often prefer not to conduct stillbirth surface exams and may require support and training to highlight the clinical importance of identifying and recording potential congenital anomalies. In addition, autopsies are rarely conducted in stillbirths. Very few surveillance systems have the capacity to include miscarriages or medical/elective pregnancy termination, and birth defects are likely to be missed in these cases. Surveillance approaches may also need to be augmented with social science research and other research to assess the impact of the introduction of PrEP on stigma, health-seeking behaviour, fertility, risky sexual behaviour and the rates of other STIs.

Longer term studies aimed at assessing the effect of in-utero exposures on growth and neurodevelopment will be important given that such studies are lacking even in cases where there are early studies suggesting a potential risk (61–63).

A sustainable surveillance model which integrates health system strengthening is central to improved quality and monitoring of care for pregnant women. Periodic reporting from surveillance systems and feedback to health care workers accompanied by relevant training is likely to build confidence in providing the necessary care. Such data may also be required by regulators, particularly where clinical trial data is limited, to expand labelling of drugs to include pregnant and breastfeeding women, subsequently increasing access. Resources are required for such surveillance systems as they are usually not implementable within the confines of a busy, already over-burdened health care system; external funding is almost always required. It may be possible for the cost of surveillance systems to be reduced over time as these outcome measure become standard of care and with increasing digitisation of health records, making them more sustainable within the routine health care system. However, this usually occurs sometime after implementation and does not completely negate the need for ongoing surveillance support.

## 4. Approaches to safety surveillance

Below we describe passive and active surveillance systems that could be considered for PrEP safety surveillance, exploring their strengths and limitations as safety surveillance and teratovigilance methods (summarised in Table 1) with some discussion on how these relate to PrEP surveillance (64). The decision regarding which system/s to choose depends on what the key risk drivers are likely to be based on available evidence, knowledge gaps, feasibility of implementation and end-user preference. Consideration of the primary objectives of the surveillance system should made by key stakeholders including regulatory authorities, HIV/AIDS and Maternal and Neonatal Health departments, academic researchers, and pregnant women themselves.

**Table 1:** Description of different surveillance types, examples, advantages and disadvantages.

Type of surveillance	Example	Approach	Passive/active	Advantages	Disadvantages
Case reports, Medicines information systems, Pharma- driven Registries	Vigibase (65) OTIS (66) and ENTIS (67) Medicines Information Centre SA (68) Antiretroviral Pregnancy Registry (69)	Voluntary reporting of adverse events by clinical staff to a central body	Passive	Detection of signal for congenital anomalies or other adverse outcomes, potential to detect miscarriage.	Sample size usually small, denominator uncertain, difficult to quantify extent of risk, reporting bias
Hospital-based surveillance	Tsepamo (46) Eswatini (70) Uganda and Malawi Birth Defects Surveillance projects (74)	Data collection on pregnancy, exposures and outcomes, + - consented photographs of congenital anomalies, routine case record review + - interview of mothers	Active	Large cohort, comparator/control groups, good quality data when coupled with health system strengthening	Missed miscarriage, home delivery, reliant on accuracy of maternal records
Case-control studies	National Birth Defects Prevention study (71)	Matched control group without the outcome of interest enrolled with group where infants born with outcome of interest. Exposures and any other potential risk factors captured from each group and compared	Active/Passive	Detailed data on specific defects Indication of risk for factors associated with outcome, information bias, information on outcomes not included in the case definition may be limited	Small cohort depending on number of facilities involved, may not be generalisable to different socio-economic, environmental circumstances
Prospective cohort studies	Ubomi Buhle (SA) (72) Western Cape Pregnancy Register (73, 74)	Prospective collection of data from first ANC visit, through pregnancy and outcome	Active	Health system strengthening focus to improve exposure history, outcome ascertainment, embedded in routine care	Time-consuming, additional resources required, may miss miscarriages, data quality dependant on maternal record
Healthcare Data Bases	Western Cape Provincial Health Care Data Base (74)	Clinical records including laboratory tests and other specialist investigations collected electronically as part of standard of care	Active	Large, representative cohort, Data linked to pharmacy dispensing records, laboratory results, specialist services, using unique identifier	Date of conception and gestational age usually unknown Challenges in controlling for bias and confounding

### 4.1. Passive surveillance reporting systems

#### 4.1.1. Case reports (spontaneous reporting)

Spontaneous reporting systems of individual case reports of suspected adverse reactions are a standard pharmacovigilance approach applied globally. These regulatory systems are an inexpensive but effective way of monitoring the safety of all health products, enlisting the support of health professionals and the public to provide information on the safety performance of these products in the country. In recent years, systems have been digitised allowing for easier and more timely reporting. Important signals related to teratogenic exposures have been detected through the reporting of individual clinical case reports. The risk of phocomelia and other major limb malformations with thalidomide, the teratogenic risk of isotretinoin and mycofenolate in pregnancy are well-known examples of teratogens identified through case reports and spontaneous reporting (75). However, the system depends on *voluntary* registration of events by clinical staff, and underreporting and reporting biases remain key challenges affecting the reliability of the data. In the case of pregnancy-related events, the delay between the timing of exposure during pregnancy and manifestation of the adverse outcome at birth, confounded by events and other exposure in-between means that spontaneous reporting has some limitations as a signal detection tool in pregnancy exposure cases. Nevertheless, it remains a useful tool for maternal adverse reactions and adverse reactions that WOCP may encounter. In addition, given the lack of a denominator and a reliable comparator group, spontaneous reporting is not able to accurately assess the magnitude of risk. Nevertheless, efforts are underway to optimise spontaneous reporting forms to collect better data from pregnancy-related reports (76).

#### 4.1.2. Medicines/teratology information centres

Medicine information centres are a valuable resource to support health care professionals with therapeutic decision-making. In high income countries, bespoke centres are in place to support pregnant and breastfeeding women and their clinicians with therapeutic decision-making in pregnancy. Teratology information centres leverage the opportunity of clinical enquiries to support collection of data on pregnancy exposures to medicines that are poorly studied. After obtaining an initial query about the safety of a particular medicine in pregnancy, the healthcare workers or the patient are contacted post-partum to determine additional pregnancy exposures and the birth outcome, including information on the presence of any birth defects. These teratology information centres are often based in academic institutions or at health facilities, are usually independent of the pharmaceutical industry and are supported by clinicians and researchers with relevant expertise in the area. That these cases are identified before the birth outcome is known and are followed up prospectively, minimizes the likelihood of recall bias. The Organization of Teratology Information Specialists (OTIS) and European Network of Teratology Information Services (ENTIS) have pooled case reports to create a “control pool” of cases of women who have been exposed to a non-teratogenic substance (67, 77). This control pool is used to conduct risk analyses for women exposed to products with an unknown risk profile (78).

Limitations include, certain information such as failed elective termination of pregnancy, self-prescribing or illicit drug use may not be disclosed/captured by these centres and there may be a bias towards more motivated responders having higher education status and more social stability resulting in selection bias. The latter may also be an advantage as data on other exposures, for example over the counter (OTC) and herbal medications, can be collected. This approach was used to identify the increased risk of birth defects with methotrexate use (79). Unfortunately, medicines information centres are currently scarce in HIV-endemic countries, with only South Africa having a maturely developed information resource in the SSA region, hence limiting the opportunities for leveraging these resources for PrEP surveillance. The Medicines Information Centre in South Africa, however is only accessible to health professionals and does not interact directly with the public (68).

### 4.2. Exposure registries managed by the pharmaceutical industry

An example of such a registry is the Antiretroviral Pregnancy Registry (APR) established in 1989, mainly for exposure to ART as treatment in pregnant women living with HIV, but also includes PrEP exposures and is still ongoing (69). This registry type is established by the manufacturers for specific drugs/drug classes such as antiretrovirals or anti-epileptic agents and are usually global (80). The APR requires voluntary enrolment of women receiving a specific ART or combination ART, resulting in a case collection. This may assist with signal detection for specific drug exposures and may be a regulatory requirement in some circumstances.

Limitations include the lack of background rates of adverse outcomes from the source population, selection bias, low levels of enrolment, particularly from LMIC settings, frequent missing data, and difficulty ascertaining risk due to the lack of background rates or comparator groups. In some instances, manufacturers create a pregnancy exposure registry for an individual product rather than a class of products. Such registries are unlikely to provide adequate data to identify signals or the controlled data needed to estimate risks of harm.

### 4.3. Hospital-based surveillance of birth outcomes

Data are collected from the maternal records or other maternity registers at pregnancy outcome in high volume delivery facilities, with a specific focus on capturing exposures and outcomes related to pre-conception (where possible) and pregnancy. These systems use records designed for recording of pregnancy-related information such as gestational age, exposures, comorbidities, as well as infant surface examination with photographs if there is a birth defect and consent is given. Examples of such surveillance systems focussing on ART include the Tsepamo study in Botswana (48) (discussed previously), ViiV study in Eswatini and the Ugandan and Malawi Birth defect surveillance programmes (70, 81, 82). The advantages of such systems are that large numbers of records of exposures and pregnancy, maternal and infant outcomes can be collected with good ascertainment of variations in frequency of birth outcomes. These surveillance models usually collect data on *all* women seeking perinatal care including HIV-exposed and unexposed women, ART exposure with different drugs (treatment and prevention) and without drug exposure, providing concurrently enrolled control groups for various risk analyses. These surveillance projects are usually conducted in a specific country or region and may not be representative of countries/regions where there are programmatic variations, socioeconomic, geographic, ethnic, or genetic variations. This model requires reliable, accurate and consistent capture of drug exposure data as part of routine maternal care and is best augmented with concurrent health system strengthening initiatives targeting data collection on medicines use so that data on exposures are elicited or captured reliably. Missing outcomes can occur in situations such as miscarriage, early stillbirths, elective and medical termination of pregnancy and home-based deliveries, although this is a limitation of most surveillance models.

### 4.4. Case control surveillance

Case control studies involve the collection and comparison of data on exposures and risk factors on infants born with the outcome of interest against similar data on an appropriately matched control group without the outcome of interest. Cases are usually derived from a number of hospitals or facilities where birth defect surveillance is being conducted. Matched controls are then selected using birth registration or hospital records to evaluate whether there are any associated risk factors for particular birth defects including data on medicine exposures. Data are collected retrospectively (e.g., by telephonic interview with the mother and/or health care provider) after delivery and includes pregnancy, family and obstetric history, medical care, diet, lifestyle, and medicine used during pregnancy. The potential pitfalls of this approach are recall bias of drug exposures history and compromised accuracy with respect to the timing of exposure as this information is elicited retrospectively. National Birth Defect Notification systems can be leveraged to identify relatively rare malformations, using matched controls from the reporting institutions. With the development of the Global Birth Defect Detection and Coding App, birth defect cases can be collected across multiple sites across the globe using a single system facilitating remote pooled coding and assessment of all cases (83).

### 4.5. Prospective cohort studies

Pregnant women are enrolled prospectively, and data are collected from their first antenatal clinic visit onwards. As with the other active surveillance approaches, this model works best when combined with health system strengthening and capacity building initiatives aimed at ensuring that maternal care and record-keeping are linked across facilities, that some identifier such as a sticker placed on the clinical record makes participants easily identifiable and that clinical record-keeping is as complete and as accurate as possible. The approach works best when rates of facility-based delivery are high, referral pathways are well-defined and clinical record-keeping during antenatal and perinatal care is linked. Gestational dating during the antenatal period and at the time of delivery allows the determination of accurate timing of exposures of interest and the assessment of prematurity as a birth outcome. Data on confounding factors for the outcome of interest including additional exposures and risk factors for adverse outcomes should be systematically collected. This prospective model requires investment in data capture embedded at sentinel sites and training and mentorship of facility staff to support high quality clinical record-keeping. This approach is time-consuming, functions best in a reasonably well-functioning health system, and benefits from the use of unique patient identifiers to facilitate record linkage and reduce loss to follow-up between enrolment and pregnancy outcome. With this approach, miscarriages, medical or elective abortions that occur after the first antenatal visit and stillbirths are less likely to be missed compared to hospital-based studies. An example of this is the Western Cape Pregnancy Exposure Registry linked to the UBOMI BUHLE pregnancy exposure registry project in South Africa (72, 73).

### 4.6. Healthcare databases

This mechanism is usually integrated within relatively sophisticated routine state or private health care information systems, where a unique patient identifier allows for all data from each patient receiving care and treatment to be linked electronically into a single patient record. There is no specific focus on a particular life period, such as pregnancy or breastfeeding, and these periods may not be accurately be ascertained from the data. Data include longitudinal follow-up and tracking through different life and health stages and across different health facilities. In these databases, maternal and infant records are linked allowing for ongoing assessment of infant outcomes beyond birth into childhood which is advantageous. Reliable exposure ascertainment using electronic prescription and dispensing records is possible. Accurate coding of pregnancy and birth outcomes is essential, and this mechanism provides large numbers of pregnant women and their infants who have been exposed and unexposed to an infection such as HIV, with or without ART, with basic information on outcomes (e.g., Caesarean Section, livebirth, stillbirth, birth weight, maternal and neonatal death). Data on exposure to over-the-counter, complementary and traditional agents and medicines dispensed via ward stock may be missing (84) as well as data on outcomes of home-based deliveries and miscarriages. Gestational age and conception dates are often missing and require computational estimation of timing of exposure, which may limit accuracy of the timing of exposure related to pregnancy or breastfeeding. Depending on the health system in question, outcomes that require higher levels of expertise and diagnostic capacity such as specific congenital malformations may be inaccurate, incomplete or missing. An example of such a platform is the Western Cape Provincial Health Data Centre in South Africa, which has already been used to assess the safety performance of isoniazid preventive therapy in women with HIV (74).

## 5. Longer term safety outcomes

A limitation of all current surveillance systems is not extending through to the postnatal and breastfeeding period and longer term, to assess growth and neurodevelopmental effects in childhood following in-utero exposures (85). Existing and future cohort studies to assess the growth and development of HIV-exposed and HIV-unexposed uninfected infants could be leveraged to assess the effect of PrEP exposures in pregnancy and breastfeeding. Healthcare databases are often able to link maternal and infant records allowing for longer term follow-up of exposed infants into childhood as well as detecting adverse birth outcomes and congenital disorders only identified in infancy and childhood (e.g., through linkage to paediatric cardiology, surgery and renal services). Studies assessing growth and neurodevelopment need to use measurement tools and approaches that have been validated in the local population while ensuring that data can be pooled across sites and settings.

## 6. Surveillance during breastfeeding

Most of the approaches above do not focus on safety issues that may arise in the infant as a result of breastfeeding alone. Very often, exposures to medicines occurs as a continuum from pregnancy into the breastfeeding period. Early infancy is fraught with confounding factors including the coincidental manifestation of infections and underlying clinical conditions. For this reason, attribution of causal association between exposure of a medicine during breastfeeding and the occurrence of an adverse effect is challenging and complex. Exposures are also difficult to measure, particularly with reliance on maternal history of breastfeeding and limited information on the extent to which medicines are excreted into breastmilk. Current knowledge suggests that accumulation of PrEP medicines in breastmilk is minimal (29). Passive surveillance systems and regular review of the international biomedical literature for signal case reports of potential harms associated with breastfeeding may be the first approach to determining the need for more active targeted surveillance approaches for infants exposed during breastfeeding.

## 7. Selecting a surveillance mechanism

Given the strengths and challenges of the various approaches outlined above, a number of factors need to be considered when developing a safety surveillance plan for PrEP products in pregnant and breastfeeding women and their children. First and foremost, strategic aims and objectives of such a plan need to be developed based on a thorough assessment of what is known and remains to be studied in terms of the safety and tolerability of PrEP products. These plans will vary according to country, region or even within-country. Perhaps most important would be consideration of the extent to which these products are likely to be used in pregnant and breastfeeding populations in the country and hence the public health importance of ensuring that these products have a favourable risk-benefit profile in the local context. Ideally, in high HIV-endemic settings, both active and passive surveillance systems should form part of the surveillance plan. In such settings, a landscape analysis could identify existing research and surveillance systems that can be leveraged to support PrEP safety surveillance. Political support for such surveillance will be critical in ensuring that the findings of such surveillance projects can inform policy. The spontaneous reporting system usually overseen by the national regulatory authority remains the mainstay of passive surveillance for all medicines including medicines used in pregnancy. The custodians of these systems need to work closely with public health researchers and policymakers engaged in developing and implementing the active surveillance system in order to ensure that signals can be detected, validated and assessed in collaboration.

The resources required to implement the chosen surveillance plan and feasibility thereof are strong considerations and include financial, implementation (for example, electronic devices, network availability), and human resources, particularly regarding how much surveillance can occur within the public health system and how much support is needed. Some models, such as a prospective surveillance system, are more costly but allow more accurate data to be collected; others may be more easily implemented within a routine public health system but produce less accurate results. The numbers of potentially eligible patients need to be considered in choosing the surveillance approach, for example in an environment where high prevalence of disease and drug use exists, it may be cost-effective to select a prospective or hospital-based surveillance system whereas in a lower prevalence environment, data could contribute to a global registry such as the APR. There is growing appreciation of the need for a signal surveillance platform to assess a variety of exposures in pregnant women and infants rather than bespoke projects looking at specific drugs or clinical conditions. A surveillance system may include a hybrid of high quality (such as prospective) and cross-sectional approaches (such as hospital-based or case-collection), ensuring that data can be compared in order to improve our understanding of the findings while expanding the data sources on which policies will be based. As far as possible, aligning surveillance system data points between different projects and geographical areas will allow later analysis across these diverse areas, increasing the generalisability of signals or risk factors across different populations.

## 8. Conclusions

As options for PrEP products, and access increases in WOCP, pregnant and breastfeeding women, pharmacovigilance systems that encompass both active and passive surveillance, provide an important opportunity to monitor the safety of current and new PrEP products in women and their infants. These surveillance systems also provide reassurance to both public health programmes, clinicians, and clients, that efforts are underway to ensure that recommended PrEP products have a favourable risk-benefit profile based on robust evidence. In establishing surveillance systems in-country, existing systems should be identified and strengthened, and there should be coordination across systems in-country regarding data triangulation, involvement of relevant stakeholders, avoiding duplication of new initiatives and allowing the design and implementation of new systems to be relevant and informed by experts.

## Data Availability

The raw data supporting the conclusions of this article will be made available by the authors, without undue reservation.
